# Clear cell adenocarcinoma of the uterine cervix in an 18 year-old pregnant female^☆^

**DOI:** 10.1016/j.gynor.2013.04.007

**Published:** 2013-05-07

**Authors:** Erin Ashton, Amy Brown, James Hoffman, Seema Khutti

**Affiliations:** a Department of Obstetrics and Gynecology, University of Connecticut Integrated Residency Program, Farmington, CT, USA; b Department of Gynecologic Oncology, Hartford Hospital, Hartford, CT, USA; c Department of Gynecologic Oncology, The Hospital of Central Connecticut, New Britain, CT, USA; d Department of Pathology, Hartford Hospital, Hartford, CT, USA

**Keywords:** Clear cell adenocarcinoma, Cervix, Pregnant, Pap smear

## Abstract

• An 18 year old nulliparous woman was diagnosed with clear cell adenocarcinoma of the cervix in pregnancy.
• LEEP conization at 33.3 weeks gestation led to premature rupture of membranes. Following cesarean section, a radical hysterectomy was performed.

## Introduction

Clear cell adenocarcinoma of the uterine cervix is a rare tumor accounting for only 4% of adenocarcinoma of the cervix (Hiroyuki et al., 2003). Cervical cancer is the most common gynecologic malignancy encountered in pregnancy. Overall, up to 3% of cervical cancers are diagnosed during a pregnancy (Nguyen et al., 2000). Despite the relative frequency of cervical cancer in pregnancy, there has been a dearth of prospective trials to determine the proper management of this circumstance, and we have found only one published report of clear cell carcinoma in pregnancy. It is a common practice to delay diagnostic surgery until the second trimester, and where possible, to delay definitive treatment until maturity of the infant.

## Case

A healthy, 18 year-old G2P0010 presented at 6 weeks gestation for her initial prenatal visit. Routine screening for HIV and other sexually transmitted infections was negative. She had no prior Pap smears. A screening Pap smear was performed which showed atypical glandular cells (AGC) suspicious for neoplasia, and a diagnosis of endocervical carcinoma was favored by the pathologist. A repeat Pap smear was obtained at 12 weeks gestation which again showed atypical endocervical cells favoring neoplastic changes, which were concerning for adenocarcinoma. Screening for high risk HPV was negative, and no lesion was visible on colposcopy. Despite the usual recommendation against endocervical curettage (ECC) during pregnancy, this procedure was cautiously done at 15 weeks gestation, finding only mucous and inflammatory cells, but no identifiable epithelium.

**Fig. 1:**
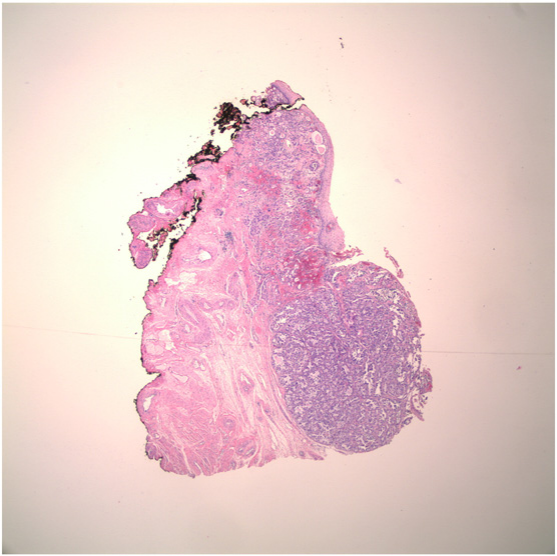
Low power view (20 ×) of cervical lesion with clear illustration of positive endocervical margin.

**Fig. 2:**
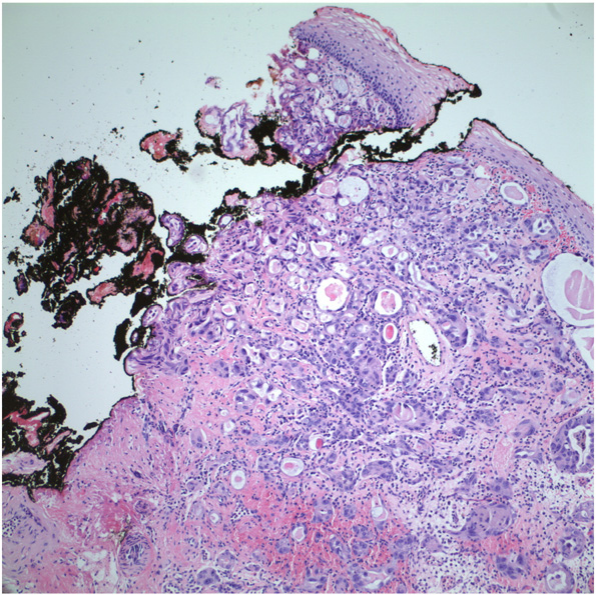
High power view (200 ×) of positive margin from the LEEP specimen. Slides submitted by Seema Khutti, MD.

Cervical conization was recommended, but was declined by the patient. Magnetic resonance imaging was performed, with no signs of cervical mass, parametrial disease, or lymphadenopathy. The patient declined further evaluation until 31 weeks gestation, when she had a directed biopsy of a clinically suspicious lesion. Pathology showed adenocarcinoma in situ, but was again concerning for invasive adenocarcinoma. An ECC also showed atypical glandular material. Her obstetrician then performed a LEEP biopsy at 33.3 weeks gestation, which revealed clear cell carcinoma of the cervix. Depth of invasion was documented to be 2 mm, but the endocervical margin was involved (see Figs. 1 and 2). There was no evidence of lymphovascular invasion. The LEEP procedure was complicated by premature rupture of membranes. At this point, the patient agreed to consultation with both gynecologic oncology and maternal fetal medicine. After corticosteroid administration for fetal lung maturity, a decision was made to perform a low transverse Cesarean section at 34 weeks gestation. Following delivery of a live infant, a radical hysterectomy with pelvic and para-aortic lymph node dissection, and bilateral oophoropexy was performed. The final pathology showed clear cell adenocarcinoma, stage IB (pT1b1, pN0, cM0). Tumor depth in the hysterectomy specimen was approximately 2 millimeters and tumor breadth approximately 5–6 millimeters. There was no evidence of metastasis to the lymph nodes or of lymphovascular invasion. The patient did not receive adjuvant therapy following hysterectomy.

Internal examinations and Pap smears of the vaginal cuff have been done at regular intervals for the ensuing three years, and have remained normal.

## Discussion

Recent guidelines of the American Society for Colposcopy and Cervical Pathology (ASCCP) have stressed that women should not receive Pap cytology testing until age 21 (Saslow et al., 2012). The expressed concern is that abnormal Pap cytology findings in younger women will lead to unnecessary interventions and an increased risk of pregnancy complications. They present data to show a high probability that many lesions in young women would have regressed spontaneously, or are usually “many years from having significant potential for becoming cancer” (Saslow et al., 2012). They refer to the increased risk of pregnancy complications and preterm delivery, and speculate that the net harm probably exceeds the benefit.

The ASCCP recommendation does not use the age of sexual debut as a criterion for when to begin cytologic screening, though they do note that increased screening may be needed in immunocompromised or HIV positive patients. Neither of these factors would have applied to the patient in this report. Other authors have expressed the opinion that some populations of young women might benefit from early screening. In an observational study on adolescents who were referred to a colposcopy clinic in the United Kingdom over a 10 year period, Saleh et al. (2007 Nov) recognized an increased incidence of moderate or severe dyskaryosis in those whose first sexual contact was before the age of 16, and in those with multiple sexual partners. These authors concluded that younger women should remain in the screening population.

Our case preceded the current ASCCP guidelines, and it is interesting to speculate on the differences in outcome that the new guidelines might have engendered. Without an early pregnancy Pap smear, we would probably not have detected this asymptomatic, early adenocarcinoma prior to delivery. She would not have had the LEEP conization at 33 weeks, with the ensuing premature rupture of the membranes and subsequent pre-term delivery. Perhaps her cancer would have been visually detected on a post-partum exam, or clinically suspected from symptoms. A conization at that time could have been sufficiently radical to allow complete assessment of her cervix. This would have averted the necessity to choose a surgical procedure before we had complete information about her cancer. Conversely, it is possible that her cancer would not have been found post-partum or might have progressed quickly given the more aggressive clear cell histology. In that case, she might have faced the hazards of a more advanced cervical cancer. Our case illustrates both the potential harms and the potential benefits that were considered by the ASCCP prior to their recommendation on the age at which screening begins. In balance we find that it is not possible to fully endorse the management of this case. LEEP conization at 33.3 weeks led directly to an early delivery and the shallow nature of this specimen prevented an adequate understanding of her pathology. If the size of her lesion had been fully evaluated, this would have potentially allowed consideration of fertility sparing surgery.

Dargent et al. (1994) first described “fertility sparing” trachelectomy for women with cervical cancers less than 2 cm. Radical vaginal, laparoscopic, and abdominal trachelectomy with laparoscopic lymph node dissection have all been reported. At the time of this patient's presentation, the literature to support fertility sparing trachelectomy was still limited; with Abu-Rustum reporting on 42 patients and Plante on 72 patients (Abu-Rustum et al., 2006; Plante et al., 2005). The majority of patients in both of these studies had squamous cell carcinoma and there was no specific experience with clear cell carcinoma. Shortly after this patient's delivery, Ramirez et al. (2008) reported on 520 patients who had radical trachelectomy, and found a 50% rate of term birth among those patients who attempted pregnancy.

Since that time, however, there has been significant increase in the literature supportive of both the oncologic safety and reproductive outcomes of fertility sparing treatment. Even in their updated series, Plante in 2011 and Abu-Rustum in 2012 with a combined total of 245 patients included no cases of clear cell carcinoma (Plante et al., 2011; Kim et al., 2012).

Lack of data regarding the safety of conservative management with the more aggressive clear cell histology along with uncertainty as to the extent of disease led us to favor radical hysterectomy rather than conservative management in our patient. The outcomes for both the mother and baby have remained excellent. The subsequently published data regarding the safety of extending the applications of fertility sparing surgery, and the policy change brought about by the recommendation of the ASCCP to stop Pap screening in teenage girls both suggest that it is important to consider alternative methods of managing early cervical cancer in future pregnant patients.

## Conflict of interest statement

None of the authors have any actual or potential conflict of interest including any financial, personal or other relationships with other people or organizations within 24 months of beginning the submitted work that could inappropriately influence, or be perceived to influence, their work.

## References

[bb0030] Abu-Rustum N.R., Sonoda Y., Black D. (2006). Fertility-sparing radical abdominal trachelectomy for cervical carcinoma: technique and review of the literature. Gynecol. Oncol..

[bb0025] Dargent D., Brun J.L., Roy M. (1994). La trachelectomie elargie (TE), une alternatie a l'hysterectomie radical dans le traitement des cancers infiltrants developpes sur la face externe du col uterin. J. Obstet. Gynaecol..

[bb0005] Hiroyuki S., Toshio T. (2003). A young woman with clear cell adenocarcinoma of the uterine cervix. Int. J. Clin. Oncol..

[bb0050] Kim C.H., Abu-Rustum N.R., Chi D.S. (2012). Reproductive outcomes of patients undergoing radical trachelectomy for early-stage cervical cancer. Gynecol. Oncol..

[bb0010] Nguyen C., Montz F., Bristow R. (2000). Management of stage I cervical cancer in pregnancy. Obstet. Gynecol. Surv..

[bb0035] Plante M., Renaud M.C., Hoskins I.A. (2005). Vaginal radical trachelectomy: a valuable fertility-preserving option in the management of early-stage cervical cancer: a series of 50 pregnancies and review of the literature. Gynecol. Oncol..

[bb0045] Plante M., Gregoire J., Renaud M.C. (2011). The vaginal radical trachelectomy: an update of a series of 125 cases and 106 pregnancies. Gynecol. Oncol..

[bb0040] Ramirez P.T., Schmeler K.M., Soliman P.T. (2008). Fertility preservation in patients with early cervical cancer: radical trachelectomy. Gynecol. Oncol..

[bb0020] Saleh M.M., Seoud A.A., Zaklama M.S. (2007 Nov). Study of the demographic criteria and management of adolescents referred with abnormal cervical smears. J. Obstet. Gynaecol..

[bb0015] Saslow D., Solomon D., Lawson H. (2012). American Cancer Society, American Society for Colposcopy and Cervical Pathology, and American Society for Clinical Pathology screening guidelines for the prevention and early detection of cervical cancer. CA Cancer J. Clin..

